# Systematic review of efficacy, safety and pharmacokinetics of intravenous and intraventricular vancomycin for central nervous system infections

**DOI:** 10.3389/fphar.2022.1056148

**Published:** 2022-11-18

**Authors:** Shu-Ping Liu, Jing Xiao, Ya-Li Liu, Yue-E Wu, Hui Qi, Zhuang-Zhuang Wang, A-Dong Shen, Gang Liu, Wei Zhao

**Affiliations:** ^1^ Key Laboratory of Major Diseases in Children, National Center for Children’s Health, Ministry of Education, Department of Infectious Diseases, Beijing Children’s Hospital, Capital Medical University, Beijing, China; ^2^ Key Laboratory of Major Diseases in Children, Beijing Key Laboratory of Pediatric Respiratory Infection Diseases, National Clinical Research Center for Respiratory Diseases, Ministry of Education, National Key Discipline of Pediatrics (Capital Medical University), Beijing Pediatric Research Institute, Beijing Children’s Hospital, Capital Medical University, Beijing, China; ^3^ Center for Clinical Epidemiology and Evidence-based Medicine, National Center for Children’s Health, Beijing Children’s Hospital, Capital Medical University, Beijing, China; ^4^ Key Laboratory of Chemical Biology (Ministry of Education), Department of Clinical Pharmacy, School of Pharmaceutical Sciences, Cheeloo College of Medicine, Shandong University, Jinan, China; ^5^ Children’s Hospital Affiliated to Zhengzhou University, Henan Children’s Hospital, Zhengzhou Children’s Hospital, Zhengzhou, China; ^6^ NMPA Key Laboratory for Clinical Research and Evaluation of Innovative Drug, Qilu Hospital of Shandong University, Shandong University, Jinan, China

**Keywords:** vancomycin, central nervous system, infections, efficacy, safety, pharmacokinetics

## Abstract

**Objective:** The decision of vancomycin dosage for central nervous system (CNS) infections is still a challenge because its bactericidal nature in cerebrospinal fluid (CSF) has not been confirmed by human studies. This study systematically reviewed the literatures on vancomycin in patients with meningitis, ventriculitis, and CNS device-associated infections, to assess efficacy, safety, and pharmacokinetics to better serve as a practical reference.

**Methods:** Medline, Embase, and Cochrane Library were searched using terms vancomycin, Glycopeptides, meningitis, and central nervous system infections. Data were extracted including characteristics of participants, causative organism(s), administration, dosage, etc., The clinical response, microbiological response, adverse events and pharmacokinetic parameters were analyzed.

**Results:** Nineteen articles were included. Indications for vancomycin included meningitis, ventriculitis, and intracranial device infections. No serious adverse effects of intravenous (IV) and intraventricular (IVT) vancomycin have been reported. Dosages of IV and IVT vancomycin ranged from 1000–3000 mg/day and 2–20 mg/day. Duration of IV and IVT vancomycin therapy most commonly ranged from 3–27 days and 2–21 days. Therapeutic drug monitoring was conducted in 14 studies. Vancomycin levels in CSF in patients using IV and IVT vancomycin were varied widely from 0.06 to 22.3 mg/L and 2.5–292.9 mg/L. No clear relationships were found between vancomycin CSF levels and efficacy or toxicity.

**Conclusion:** Using vancomycin to treat CNS infections appears effective and safe based on current evidence. However, the optimal regimens are still unclear. Higher quality clinical trials are required to explore the vancomycin disposition within CNS.

## 1 Introduction

Central nervous system (CNS) infections, including community-acquired bacterial meningitis (CABM) and healthcare-associated meningitis and ventriculitis (HCAVM) (Giovane and Lavender, 2018; Expert Panel on Neurological et al., 2019; Bloch and Hasbun, 2021), are particularly prevalent and associated with significant morbidity and mortality (Hasbun, 2019). Gram-positive organisms are one of the main pathogens for CNS infections (Levin and Lyons, 2018; Li et al., 2018). Owing to the emergence of penicillin-resistant Gram-positive organisms, vancomycin is widely used as an empiric treatment for bacterial CNS infections (Lewin et al., 2019). The decision of vancomycin dosage for CNS infections is still a challenge for two reasons: 1) the effective therapeutic concentrations in the CNS and 2) the time to reach the target of cerebrospinal fluid (CSF) concentration (van de Beek et al., 2012; Ng et al., 2014). Although Infectious Diseases Society of America (IDSA) recommends drug concentrations exceeding the minimum inhibitory concentration (MIC) 10–20 times for consistent CSF sterilization (Tunkel et al., 2017), the ratio of minimum CSF concentration to MIC for successful treatment is still unclear (Tunkel et al., 2004; Posadas and Fisher, 2018).

Vancomycin is a high molecular weight complex glycopeptide antibiotic that has been approved for clinical use since 1958. Vancomycin inhibits cell wall synthesis of bacteria by forming stable complex murein pentapeptide (Jacqz-Aigrain et al., 2019). Vancomycin exhibits time-dependent bacterial killing in serum (Rybak et al., 2020). But Vancomycin’s time-dependent bactericidal nature has not been confirmed in CSF. Current most studies suggested that penetration of vancomycin in the CNS is limited partly because of its hydrophilicity (Beach et al., 2017). Pharmacokinetic parameters of vancomycin in CSF are different from all other body sites due to the physiology of the cerebrospinal fluid. Moreover, potential device placement may alter normal physiological clearance of CSF (Ng et al., 2014). In determining the appropriate dosage strategies for vancomycin, its unique pharmacokinetic and pharmacodynamic characteristics in CNS infections must be considered (Hoen et al., 2019).

Unfortunately, few clinical trials performed for appropriate dosage of vancomycin in CNS infections have been published to guide use in routine clinical practice. In order to serve as a practical reference, we systematically reviewed the current literatures on intravenous (IV) and intraventricular (IVT) vancomycin in treatment of CNS infections. Where available, pharmacokinetic and pharmacodynamic (PK/PD) data were also summarised.

## 2 Materials and methods

### 2.1 Protocol and guidelines

The study was conducted and presented in accordance with the Preferred Reporting Items for Systematic Reviews and Meta-Analyses (PRISMA) statement (Moher et al., 2015) and the Synthesis Without Meta-analysis (SWiM) guideline (Campbell et al., 2020). The systematic review protocol was not published.

### 2.2 Search strategy

The databases MEDLINE, EMBASE and Cochrane Library were searched for evaluating vancomycin in therapy for CNS infections, using the terms “vancomycin” (MeSH) OR “vancomycin” (Title/Abstract) OR “Glycopeptides” (Title/Abstract) AND “meningitis, bacterial” (MeSH) OR [“nervous system diseases” (Title/Abstract) OR “meningit*” (Title/Abstract) OR “central nervous system infections” (Title/Abstract)] for articles. Searches were limited to articles published in English up to 24 July 2020. Titles and abstracts were manually reviewed. Reference lists were also manually searched for the relevant articles.

### 2.3 Eligibility criteria

Any published literature with documented involvement of patients administered vancomycin *via* any route of administration for CNS Infections was reviewed.

Inclusion criteria: 1) Patients: confirmed CNS Infections by laboratory, including meningitis, ventriculitis, and CNS device-associated infections; 2) Intervention: treated with vancomycin; 3) Comparison for pharmacodynamic analysis: patients in the control group were given modern conventional treatments; 4) Outcomes: clinical efficacy or safety or of vancomycin for CNS Infections, therapeutic drug monitoring of vancomycin, or pharmacokinetic parameters; 5) Study types: Randomized controlled trials (RCTs), nonrandomized controlled trials, cohort studies, case-control studies (CCSs), cross-sectional studies or pharmacokinetic studies. We excluded the following studies: 1) studies focusing on neurosurgical prophylaxis; 2) studies not focusing on vancomycin; 3) case reports, reviews, animal studies, letters, comments, abstracts, and editorials.

### 2.4 Study selection and data extraction

All results were reviewed independently by two investigators (S-PL and JX), any controversial item was resolved through discussion and adjudicated by the third author (Y-LL). Data collection were conducted independently by two authors (S-PL and JX) with a standardized approach. Data were extracted from the relevant articles on methodology, characteristics of trial participants (including age, gender, and indication), causative organism(s), number of patients receiving vancomycin, number of participants in study, route of administration, dose of vancomycin, therapeutic drug monitoring, treatment duration, clinical response, microbiological response, adverse events and pharmacokinetic parameters.

### 2.5 Quality assessment

Studies were assessed by two reviewers (S-PL and JX) using the Risk of Bias (RoB) assessment tool from the Cochrane Handbook for RCTs (Higgins et al., 2011), and the Newcastle-Ottawa Scale (NOS) for CCSs (Stang, 2010). All included pharmacokinetic studies or studies containing evidence regarding therapeutic drug monitoring and dosing were evaluated by two authors (S-PL and Y-EW.) using the 24-item ClinPK statement checklist (Kanji et al., 2015).

### 2.6 Statistical analysis

Stata (version 13.0; StataCorp) and Review Manager 5.3 were used to perform the statistical analysis. Risk ratio (RR) was used for dichotomous data. Effect size was expressed as weighted mean difference (WMD) and 95% confidence intervals (CI). Considering heterogeneity was calculated based on the random effect model. *p*-value less than 0.05 indicated significant statistically differences. The limited data were inadequate for a meta-analysis of efficacy or safety and therefore a descriptive analysis were performed, according to the SWiM guideline.

## 3 Results

### 3.1 Flow and characteristics of included studies

**FIGURE 1:**
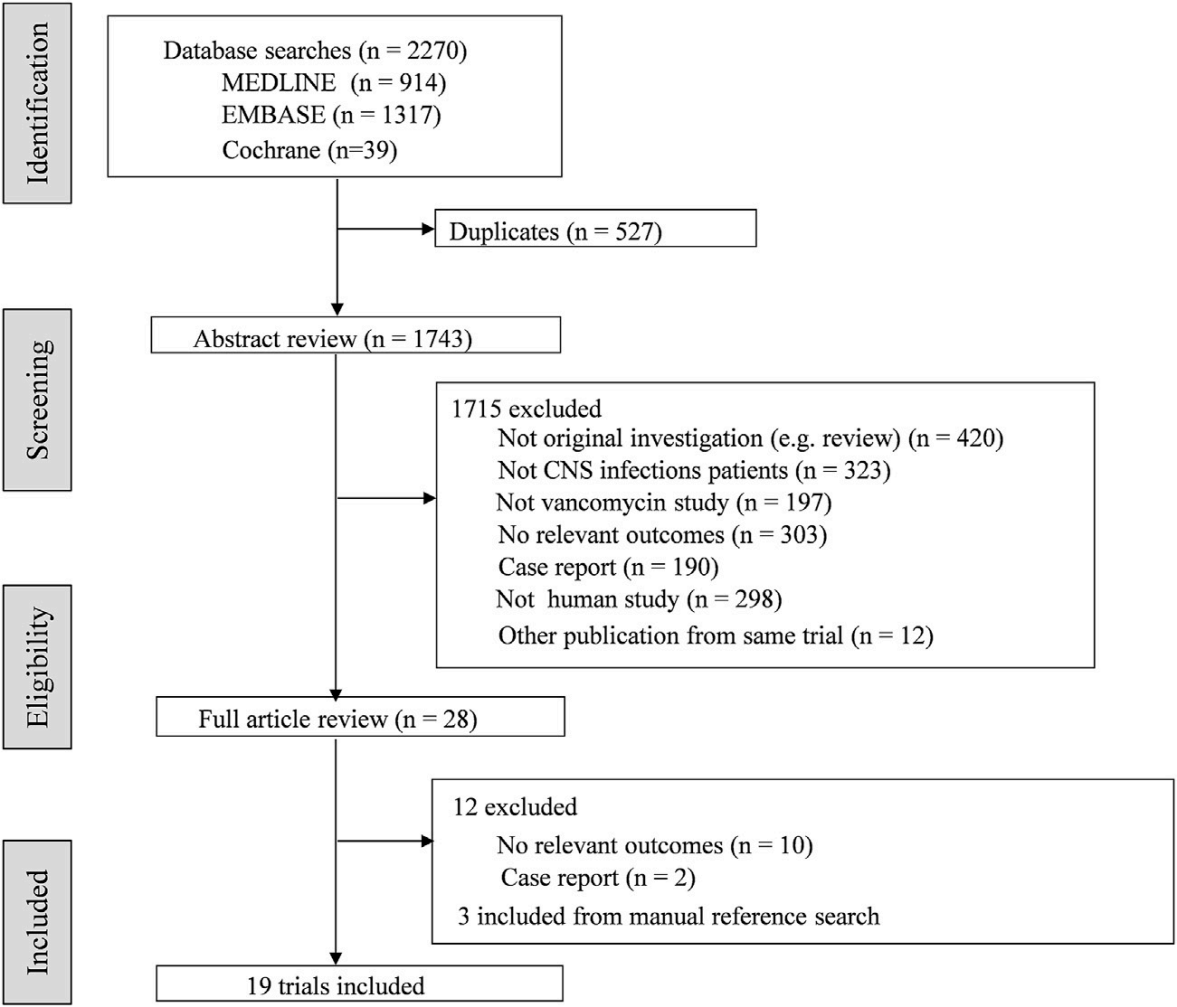
Flow chart of literature search and selection process.

A total of 19 articles involving 482 patients were identified (Figure 1). The characteristics of included studies were summarized in Table 1. In general, 6 studies involved patients treated with IVT vancomycin (Pfausler et al., 1997; Pfausler et al., 2003; Bafeltowska, Buszman, Mandat, and Hawranek, 2004; Popa et al., 2016; Parasuraman et al., 2018; Lewin et al., 2019), and 13 studies were IV vancomycin (Viladrich et al., 1991; Albanese et al., 2000; Arda et al., 2005; Ricard et al., 2007; Sipahi et al., 2013; Autmizguine et al., 2014; Shokouhi and Alavi Darazam, 2014; Elyasi et al., 2015; Lin et al., 2016; Mounier et al., 2017; Wang et al., 2017; Taheri et al., 2018; Cai et al., 2019). Fourteen studies were regarding pharmacokinetic analysis and dosing (Supplementary Appendix 1). Of these, 10 trials reported serum and CSF vancomycin concentrations (Viladrich et al., 1991; Albanese et al., 2000; Ricard et al., 2007; Autmizguine et al., 2014; Shokouhi and Alavi Darazam, 2014; Popa et al., 2016; Mounier et al., 2017; Wang et al., 2017; Taheri et al., 2018; Cai et al., 2019), and 6 provided CSF-to-serum ratios (Albanese et al., 2000; Autmizguine et al., 2014; Shokouhi and Alavi Darazam, 2014; Wang et al., 2017; Taheri et al., 2018; Cai et al., 2019). All trials reported the vancomycin serum or CSF sampling technique and timing. Five trials provided some information of PK Parameters (Albanese et al., 2000; Bafeltowska et al., 2004; Autmizguine et al., 2014; Lin et al., 2016; Taheri et al., 2018). Of these, only one trial described Population PK model covariates (Lin et al., 2016). Three RCTs (Pfausler et al., 2003; Elyasi et al., 2015; Taheri et al., 2018) and three case control studies (Arda et al., 2005; Sipahi et al., 2013; Lewin et al., 2019) analysed clinical or loboratory response of treatment with intravenous or intraventricular vancomycin (Supplementary Appendix 2).

**TABLE 1:** Characteristics of the trials.

Author (year)	Sample size	Age	Sex (male)	Indication	Pathogen (number)	Treatment details	TDM	
							Serum level (mg/L)	CSF level (mg/L)
Cai, (2019)	22	44.38 years (SD 14.05)	13 (59%)	PII and CAM	NA	VAN 1 g IV q12h	10	2.16 ± 1.23
Lewin, (2019)	105	49.9 years (SD 17.6)	62 (59%)	CNS infections	CoNS (23), *S. aureus* (11), K*lebsiella species* (10), and *Pseudomonas species* (6)	VAN 12.2 ± 5.8 mg IVT q24h × 5 days; Gentamicin/tobramycin 6.7 mg ± 3.4 mg and Amikacin 22.5 mg ± 3.5 mg IVT × 6 days	NA	NA
Parasuraman, (2018)	7	Gestational age: 25 + 4 weeks	NA	VPS in preterm infants	NA	VAN 3, 5, 10 and 15 mg IVT q24h × 5.5 days (range 2–31 days)	NA	3 mg IVT q24h, C_max_ = 24.9, C_min_ = 3.5; 5 mg IVT q24h, C_max_ = 96.3, C_min_ = 2.5; 10 mg IVT q24h, C_max_ = 94, C_min_ = 4.2; 15 mg IVT q24h, C_max_ = 230.7, C_min_ = 44.9.
Taheri, (2018)	20	48.5 years (SD 7.46)	11 (55%)	PNM	*A. baumannii* (1), MRSA (1) and *P. aeruginosa* (1)	II: VAN 25 mg/kg IV q12h; CI: a loading dose of VAN 25 mg/kg IV over 2 hours, followed by 50 mg/kg daily by continuous infusion	C_min_ = 17.49 ± 2.46, C_max_ = 41.33 ± 2.73, Caverage 24.76 ± 2.02	5.52 ± 1.35
Mounier, (2017)	6	43 years (SD 14.3)	4 (67%)	VPS	*Staphylococcus*	VAN 60 mg/kg IV daily after a loading dose of 15 mg/kg	C_min_ = 35.61 ± 21.51	1.00 ± 1.03
Wang, (2017)	22	52.6 years (SD 12.1)	14 (64%)	PNM	*S. pneumoniae* (1), *E. faecium* (1), *S. aureus* (1), CoNS (1), *S. saprophyticus* (1) and *E. hirae* (1)	VAN 500 mg IV q6h for alone or in combination with Ceftriaxone 2 g IV bid	C_min_ = 13.38 ± 5.36	3.63 ± 1.64
Lin, (2016)	120	Range, 18–86 years	79 (65.83%)	PCM	NA	VAN 500 mg, 750 mg, 1000 mg, 1250 mg, or 1500 mg IV q12 h	C_min_ = 10.5 ± 8.9	NA
Popa, (2016)	13	58 years (SD 29.8)	8 (62%)	Meningitis	VS. + *N. mucosa* + GH (1), EC + *A. aphrophilus* + CoNS (1), *S. aureus* + CoNS (1), *S. pneumoniae* (2), *S. anginosus* (1), *S. aureus* (2), EM (1), CoNS (1), and GAS (1)	VAN 33.3 ± 14.5 mg/kg IV daily × (8.6 ± 7.1) days and VAN 9.3 ± 2.2 mg/kg IVT daily × (4.1 ± 2.5) days. There was an average of 2.7 days of overlap between IV and IVT therapy.	C_min_ = 18.53 ± 7.53	35.39 ± 50.09
Elyasi, (2015)	44	Range, 29–69 years	NA	BM	*S. pneumoniae* (25), MRSA (2),*S. epidermidis*, (1) and *E. faecaliss* (1)	High-dose group: VAN 15 mg/kg IV q8 h × 10 days; conventional-dose group: VAN 15 mg/kg IV q12 h × 10 days	NA	NA
Autmizguine, (2014)	8	Range, 0.2–17 years	4 (50%)	VPS	MRSA (1), MRSA + *E. coli* (1), *E. coli* (1), CoNS (1) and *Gordonia sp.*/*Rhodococcus sp.* Group (1)	VAN 19 mg/kg/dose (11–30) IV q8h (7–13) × 17 days (4–27)	C_min_ = 11.5 (3.9–32.1)	1.07 (0.06–9.13)
Shokouhi, (2014)	27	39.4 years (SD 14.7)	18 (67%)	CAM	NA	VAN 15 mg/kg loading and 30 mg/kg IV daily maintenance dose	C_min_ = 13.57 ± 1.17	10.92 ± 1.33
Sipahi, (2013)	17	61.6 years (SD 13.2)	12 (71%)	PNM and VPS	MRSA + MRCNS (1) and MRSA (9)	VAN 500 mg IV q6h × 5 days; Linezolid 600 mg IV q12h × 5 days	NA	NA
Ricard, (2007)	14	52 years (SD 20)	8 (57%)	BM	*S. pneumoniae* (13), *Neisseria meningitidis* (1)	VAN 60 mg/kg continuous IV daily after a loading dose of 15 mg/kg; cefotaxime 200 mg/kg IV daily	25.5 ± 7.3	7.9 ± 5.1
Arda, (2005)	10	34.1 years (SD 25.6)	8 (80%)	PNM and HAM	MRSA (8), MRSA + *Enterococcus spp*. (1) and MRSA + MRCNS (1)	VAN 50–500 mg IV q6-12h, teicoplanin 80–400 mg IV bid and Cefazolin 500 mg IV tid ×(23.5 ± 18.8) days	NA	NA
Bafeltowska, (2004)	10	11–151 days old	4 (40%)	VPS in children with hydrocephalus	*Staphylococcus* (4) and *E. coli* (1)	VAN 8, 20, 38 mg/kg IV daily and IVT 3–15 mg daily	NA	22.12 ± 25.66
Pfausler, (2003)	10	Range, 26–73 years	3 (30%)	VPS	CoNS sp. (8) and *S. aureus* (2)	VAN 10 mg IVT q24h × 7 days; VAN 2 g/day IV × 7 days	NA	NA
Albanèse, (2000)	13	Range, 25–58 years	NA	Meningitis	*S. epidermidis* (6), *S. aureus* (3), *S. pneumoniae* (2), *E. faecaliss* (1), and *Corynebacterium* (1)	VAN 50–60 mg/kg IV daily after a loading dose of 15 mg/kg	C_min_ = 36.24 ± 8.19, C_max_ = 22.6 ± 4.1	C_min_ = 6.20 ± 4.08, C_max_ = 11.13 ± 4.92
Pfausler, (1997)	3	>18 years	NA	VPS	MRSA (3)	VAN 10 mg IVT q24h × 5, 8, 13 days	NA	C_min_ = 7.6, C_max_ = 292.9
Viladrich, (1991)	11	40 years (SD 15)	5 (45%)	BM	*S. pneumoniae* (11)	VAN 7.5 mg/kg IV q6h ×10 days	Range 18–34	Range 4–9.4

BM, bacterial meningitis; CAM, community-acquired meningitis; CI, continuous infusion group; CoNS, *Coagulase-negative Staphylococcus*; CNS, central nervous system; EC, *Eikenella corrodens*; EM, *Elizabethkingia meningosepticum*; GAS, *Group A Streptococcus*; GH, *Gemella haemolysans*; HAM, hospital-acquired meningitis; II, intermittent infusion group; IV, intravenous; IVT, intraventricular; MRCNS, *methicillin-resistant coagulase-negative staphylococci*; MRSA, *methicillin resistant staphylococcus aureus*; NA, not available; PCM, post-craniotomy meningitis; PII, postoperative intracranial infection; PNM, post-neurosurgical meningitis; TDM, therapeutic drug monitoring; VAN, vancomycin; VPS, ventriculoperitoneal shunt infections; VS., *Viridans streptococc*i.

### 3.2 Quality of included studies

The quality assessment of the three included RCTs (Pfausler et al., 2003; Elyasi et al., 2015; Taheri et al., 2018) is shown in Supplementary Appendix 3. The quality of three case control studies (Arda et al., 2005; Sipahi et al., 2013; Lewin et al., 2019) was assessed by NOS in Supplementary Appendix 4. The study published by Lewin et al. (2019) scored 5, Sipahi OR et al. (Sipahi et al., 2013) scored 7, and Arda et al. (2005) scored 4. Each PK study or therapeutic drug monitoring study was assessed using the ClinPK statement (Supplementary Appendix 5) (Kanji et al., 2015).

### 3.3 Administration with intravenous vancomycin

#### 3.3.1 Clinical and microbiological response of intravenously administered vancomycin

• Meningitis

A single RCT was identified (Elyasi et al., 2015). During the 2-year period, 44 patients with bacterial meningitis were randomly assigned to the conventional-dose vancomycin (15 mg/kg q12 h) or high-dose vancomycin (15 mg/kg q8h) groups. In the high-dose group, leukocytosis (*p =* 0.03) and fever (*p =* 0.02) resolved significantly faster, length of hospitalization (*p =* 0.04) was shorter, and Glasgow Coma Scale (*p =* 0.02) at the end of 10th day was lower than those in the conventional group.

• Ventriculitis and Shunt Infections

A single RCT was identified (Taheri et al., 2018). Patients in intermittent infusion group (II group) received vancomycin 25 mg/kg every 12 h, and those in continuous infusion group (CI group) received vancomycin 50 mg/kg/day by continuous infusion. At the end of treatment, all patients recovered in both groups, the therapy was well tolerated. One retrospective cohort study was identified (Sipahi et al., 2013) in 17 patients with culture-proved MRSA meningitis. Of these 6 patients with vancomycin treatment failures, two died while receiving linezolid. One Patient who failed linezolid treatment died after development of *Pseudomonas aeruginosa* meningitis. Another retrospective study involved 10 MRSA post-neurosurgical meningitis cases, including 3 children (Arda et al., 2005). All patients survived except one patient. The only fatal infection was treated empirically with cefazolin and died during this treatment while awaiting the CSF culture results.

#### 3.3.2 Adverse effects of intravenous vancomycin

No adverse events were reported in these studies, including *nephrotoxicity*.

#### 3.3.3 Pharmacokinetics of intravenously administered vancomycin

Of the studies included, 13 obtaining serial CSF vancomycin concentrations post IV dose (Viladrich et al., 1991; Pfausler et al., 1997; Albanese et al., 2000; Bafeltowska et al., 2004; Ricard et al., 2007; Autmizguine et al., 2014; Shokouhi and Alavi Darazam, 2014; Popa et al., 2016; Mounier et al., 2017; Wang et al., 2017; Parasuraman et al., 2018; Taheri et al., 2018; Cai et al., 2019). Of these, 4 sought to characterize serum pharmacokinetic parameters of IV vancomycin, including 3 in adults and 1 in children (Albanese et al., 2000; Autmizguine et al., 2014; Lin et al., 2016; Taheri et al., 2018).

• Volume of distribution (V_D_)

Albanese et al. (2000) performed a serum pharmacokinetic analysis in 7 patients with bacterial meningitis that suggested that V_D_ was 0.2 ± 0.05 L/kg. For children population, a pharmacokinetic analysis performed in seven children (Autmizguine et al., 2014) showed that the V_D_ was 0.70 (0.22–4.46) L/kg in serum.

• Clearance (CL)

Lin et al. (2016) performed a prospective study of 100 adults post-craniotomy meningitis patients. A PPK model was developed using a nonlinear mixed-effect modelling program basing a one-compartment model with first-order elimination. The results showed that creatinine clearance affected vancomycin clearance. Taheri et al. (2018) evaluated serum pharmacokinetics of vancomycin in 20 post neurosurgical meningitis patients. Using a non-compartmental method, CL was 4.60 ± 0.73 L/h in the continuous infusion group and 4.86 ± 0.68 L/h in the intermittent infusion group. In another serum pharmacokinetics of vancomycin (Albanese et al., 2000), CL was 0.03 ± 0.02 L/min. The pharmacokinetic study performed in children (Autmizguine et al., 2014) found that CL was 0.08 (0.05–0.15) L/h/kg.

• Half-life (*t*_*1/2*_)

The study of serum pharmacokinetics of vancomycin (Taheri et al., 2018) found that *t*_*1/2*_ was 7.05 ± 0.89 h in the continuous infusion group and 6.99 ± 0.7 h in the intermittent infusion group. In another pharmacokinetic analysis (Albanese et al., 2000), elimination *t*_*1/2*_ was 6.9 ± 5.9 h.

#### 3.3.4 CSF penetration of intravenously administered vancomycin

In all identified studies, 6 clinical trials (Albanese et al., 2000; Autmizguine et al., 2014; Shokouhi and Alavi Darazam, 2014; Wang et al., 2017; Taheri et al., 2018; Cai et al., 2019) evaluated vancomycin CSF penetration, which CSF-to-serum ratio of vancomycin varied from 0.00 to 0.81. Most studies indicated that no factor could predict vancomycin CSF penetration. However, Albanese et al. (2000) suggested that vancomycin penetration into CSF was significantly higher in the bacterial meningitis group (48%) than in the other group (18%). Ricard et al. (2007) found a positive correlation between vancomycin penetration into CSF and the level of CSF protein. Shokouhi and Alavi Darazam, (2014) suggested that the vancomycin CSF trough concentrations were positively correlated with serum simultaneous levels (*r* = 0.71).

#### 3.3.5 Dosage regimens

Dosing regimens of IV vancomycin in reviewed studies were 1000–3000 mg/day (Viladrich et al., 1991; Albanese et al., 2000; Arda et al., 2005; Ricard et al., 2007; Sipahi et al., 2013; Autmizguine et al., 2014; Shokouhi & Alavi Darazam, 2014; Elyasi et al., 2015; Lin et al., 2016; Mounier et al., 2017; Wang et al., 2017; Taheri et al., 2018; Cai et al., 2019). A study on a low intravenous vancomycin dose of 7.5 mg/kg every 6 h for pneumococcal meningitis suggested that treatment failures occurred in 45.45% (5/11) of patients (Viladrich et al., 1991).

#### 3.3.6 Duration of therapy

Duration of therapy is highly heterogeneous between cases with an approximate range of 3–27 days (Viladrich et al., 1991; Albanese et al., 2000; Arda et al., 2005; Ricard et al., 2007; Sipahi et al., 2013; Autmizguine et al., 2014; Shokouhi and Alavi Darazam, 2014; Elyasi et al., 2015; Lin et al., 2016; Mounier et al., 2017; Wang et al., 2017; Taheri et al., 2018; Cai et al., 2019). Wang et al. suggested a 3- to 5-day treatment course for proven or highly suspected postsurgical meningitis, but 45.5% (10/22) cases required a treatment period of >5 days (Wang et al., 2017). In a prospective clinical trial in 8 children with cerebral ventricular shunt infections, bacteriologic confirmed normalization of CSF was noted after a mean duration of 17 days and no relapses were noted over a 6 month period (Autmizguine et al., 2014).

### 3.4 Administration with intraventricular vancomycin

#### 3.4.1 Clinical and microbiological response of intravenously administered vancomycin

• Meningitis

No studies.

• Ventriculitis and shunt infections

In a RCT study (Pfausler et al., 2003), much higher CSF vancomycin levels were achieved by intraventricular administration than by intravenous administration. The maximum CSF vancomycin level was 565.58 ± 168.71 μg/ml in IVT Group and 1.73 ± 0.4 μg/ml in IV Group. A retrospective study (Lewin et al., 2019) involved 44 patients who received only vancomycin. Sterilization of CSF cultures occurred in 39 out of 44 patients (88.4%) who received IVT vancomycin alone.

#### 3.4.2 Adverse effects of intraventricular vancomycin

There were no confirmed adverse effects due to the IVT treatment in the reviewed studies.

#### 3.4.3 Pharmacokinetics of intraventricularly administered vancomycin

• V_D_

A retrospective case series enrolled 13 patients who received IVT vancomycin for external ventricular drains (EVD)-related infections (Popa et al., 2016) On univariate analysis, CSF vancomycin concentrations were correlated with CSF output (*p =* 0.02) and time from dose (*p =* 0.001). Using multi-variate linear regression, only time was an independent predictor for CSF vancomycin concentration (*p =* 0.033).

• CL

As Collins described (Collins, 1983), a minimum clearance rate for all drugs is determined by ratio of CSF bulk flow to CSF volume, and is independent of properties of the drug.

• t_1/2_

Pfausler et al. (2003) found that CSF vancomycin t_*1/2*_ was extended during progression of treatment, resulting in vancomycin accumulation necessitating dosage alterations. For shunt infections in children, the *t*_*1/2*_ of vancomycin in CSF after intraventricular administration was also prolonged, ranging from 8 to 76 h (Bafeltowska et al., 2004). In contrast, Pfausler et al. (1997) did not observe vancomycin accumulation in any of 3 patients using IVT vancomycin 10 mg q24 h for over 7 days.

#### 3.4.4 Dosage regimens

Empiric dosage regimens of 5–20 mg/day are generally recommended for treating meningitis (Tunkel et al., 2004) and ventriculitis (Agrawal, Cincu, and Timothy, 2008). Empiric dosing frequency of once a day is most commonly used (Pfausler et al., 1997; Bafeltowska et al., 2004; Popa et al., 2016; Parasuraman et al., 2018). In children, the doses of IVT vancomycin used were from 2 to 20 mg (Bafeltowska et al., 2004; Parasuraman et al., 2018). A study involved 10 children with hydrocephalus shunt infections who received IVT vancomycin of doses ranging from 2 to 20 mg (Bafeltowska et al., 2004). A single-center, retrospective case series in infants reported doses ranging from 3 to 15 mg are sufficient for achieving microbiological cure and no adverse effects were observed (Parasuraman et al., 2018).

#### 3.4.5 Duration of therapy

Duration of therapy varies greatly between cases with a range of 2–31 days (Pfausler et al., 1997; Pfausler et al., 2003; Bafeltowska et al., 2004; Popa et al., 2016; Parasuraman et al., 2018; Lewin et al., 2019). A single-centre, retrospective case series (Parasuraman et al., 2018) suggested that ventriculitis resolution was achieved in a median of 5.5 days (range 2–31 days) in all included seven infants in doses ranging from 3 to 15 mg. Longer durations may be repaired in cases of fulminant ependymitis, persistent positive CSF cultures, as well as in immunocompromised patients. Source control by removing infected devices is crucial to successful bacterial eradication.

## 4 Discussion

In clinical practice, the use of vancomycin to treat CNS infections could be based on the efficacy and safety of its or other considerations. The systematic review showed that using vancomycin for CNS infections appears safe and effective. Dosages of IV vancomycin ranged from 1000–3000 mg/day and empiric dosing frequency was 15 mg/kg q6h. Dosage of IVT vancomycin were from 2 to 20 mg/day and empiric dosages were 5–20 mg/day. Vancomycin tends to penetrate CSF poorly because it is a large and hydrophilic molecule that limits passage through BBB (Beach et al., 2017). Due to the potential limitations of IV vancomycin therapy, when intravenous vancomycin does not achieve clinical and laboratory improvement in bacterial CNS infections caused by susceptible organisms that are resistant to other drugs, IVT administration may be considered (Ziai and Lewin, 2009) (“The management of neurosurgical patients with postoperative bacterial or aseptic meningitis or external ventricular drain-associated ventriculitis. Infection in Neurosurgery Working Party of the British Society for Antimicrobial Chemotherapy,” 2000; Tunkel et al., 2004).

In the reviewed studies, unexpectedly high and low CSF vancomycin concentrations have been observed (Viladrich et al., 1991; Albanese et al., 2000; Arda et al., 2005; Ricard et al., 2007; Sipahi et al., 2013; Autmizguine et al., 2014; Shokouhi & Alavi Darazam, 2014; Elyasi et al., 2015; Lin et al., 2016; Mounier et al., 2017; Wang et al., 2017; Taheri et al., 2018; Cai et al., 2019) (Pfausler et al., 1997; Pfausler et al., 2003; Bafeltowska et al., 2004; Popa et al., 2016; Parasuraman et al., 2018; Lewin et al., 2019) and successful treatment has been achieved in most cases. Trough levels are recommended to be maintained above 10–20 times the MIC of the organism (Tunkel et al., 2004); and CSF samples are to be analyzed before each subsequent dose of vancomycin (Reesor, Chow, Kureishi, and Jewesson, 1988). Brain tissue and subarachnoid space are regions where host defense is ineffective, with lacking of antibodies as well as complement in CSF (Tunkel and Scheld, 1993). Therefore, vancomycin must be dosed to reach sufficiently high concentrations to allow to eradicate infections. It is debated whether therapeutic drug monitoring (TDM) of CSF vancomycin concentrations is necessary or effective, because it is uncertain whether vancomycin is time-dependent or concentration-dependent in CSF. Additionally, the therapeutic range of CSF vancomycin concentrations has not been characterized. Therefore, routine TDM is of little value because it is unclear how it makes a significant difference in clinical decision making (Ng et al., 2014). But selective TDM may be warranted when CSF culture is not cleared after 3–5 days of treatment, duration of treatment is expected to be extended beyond 1–2 weeks, dosages are outside the usual range, or when disease states or placements and removals of devices are expected to be changing CSF physiology (Ng et al., 2014). It is recommend targeting an AUC/MIC ratio of 400–600 in both adult and pediatric patients for the treatment of serious infections to maximize clinical efficacy and minimize AKI risk (Rybak et al., 2020). A trough level of 15–20 mg/L is recommended to insure an AUC/MIC >400 in recent expert guidelines (Jeffres, 2017; Tunkel et al., 2017).

Our study had some limitations. Firstly, sample sizes are relatively small, ranging from 3 to 120 cases. Due to few CSF PK/PD data given from current evidence to guide dosing of vancomycin, optimal regimens are still unclear. Secondly, pharmacokinetic parameters of vancomycin CSF are unclear. Despite the vast amount of knowledge acquired regarding IV vancomycin in blood stream infections, these pharmacokinetic parameters cannot be applied to CNS infections because of unique differences between blood and CSF. Thirdly, it is the lack of adverse effects data. Nephrotoxicity is the most significant adverse effect. Some risk factors for vancomycin-association nephrotoxicity should be warned, such as the combination of piperacillin-tazobactam (PTZ), everity of illness, pre-existing kidney disease, and so on (Fiorito, Luther, Dennehy, LaPlante, & Matson, 2018; Abdelmessih et al., 2022).

## 5 Conclusion

Based on current evidence, using vancomycin to treat CNS infections appears safe and effective, although optimal regimens are still unclear. Dosing adjustment of vancomycin needs to consider the patient specific factors and the influence of CNS pathophysiology. Higher quality clinical trials are required to explore vancomycin disposition within CNS, so as to better characterize the PK/PD parameters and understand the effects on CNS infections.
