# Supplementary material for: Systematic review of efficacy, safety and pharmacokinetics of intravenous and intraventricular vancomycin for central nervous system infections
Source: Front Pharmacol. 2022 Nov 18;13:1056148. doi: 10.3389/fphar.2022.1056148 (PMC9718031; doi:10.3389/fphar.2022.1056148)
Supplement: Supplementary file 1 [file DataSheet1.docx]

Appendix 1. Published studies containing evidence regarding pharmacokinetic analysis of vancomycin administered intravenously or intraventricularly

| Author, year | Administration | Age Group | Penetration of vancomycin |  | PK Parameters | | | | |
| --- | --- | --- | --- | --- | --- | --- | --- | --- | --- |
|  |  |  | CSF/serum (%) |  | Ke (1/h) | Half-life (h) | Clearance (L/h) | AUC (mg × h/L) | VD (L) |
| Cai, 2019 | IV | Adult | 22±12 |  | NA | NA | NA | NA | NA |
| Taheri, 2018 | IV | Adult | 26.12±3.23 |  | 0.1±0.09 | 7.02±0.78 | 4.73±0.70 | 601.8±50.38 | NA |
| Mounier, 2017 | IV | Adult | NA |  | NA | NA | NA | NA | NA |
| Wang, 2017 | IV | Adult | 29.1±11.8 |  | NA | NA | NA | NA | NA |
| Lin, 2016 | IV | Adult | NA |  | NA | NA | 7.56 | NA | 101 |
| Autmizguine, 2014 | IV | Children | 8 (0-66) |  | 0.12 (0.02-0.26) | NA | 0.08 (0.05-0.15) L/h/kg | NA | 0.70 (0.22-4.46) L/kg |
| Shokouhi, 2014 | IV | Adult | 81.1±8.2 |  | NA | NA | NA | NA | NA |
| Ricard, 2007 | IV | Adult | NA |  | NA | NA | NA | NA | NA |
| Albanèse, 2000 | IV | Adult |  |  | NA | 6.9±5.9 | 0.03±0.02 L/min | NA | 0.2±0.05 L/kg |
| Viladrich, 1991 | IV | Adult | NA |  | NA | NA | NA | NA | NA |
| Parasuraman, 2018 | IVT | Children | NA |  | NA | NA | NA | NA | NA |
| Popa, 2016 | IV & IVT | Adult | NA |  | NA | NA | NA | NA | NA |
| Bafeltowska, 2004 | IV & IVT | Children | NA |  | NA | 8-76 | NA | NA | NA |
| Pfausler, 1997 | IVT | Adult | NA |  | NA | NA | NA | NA | NA |

Abbreviation: CSF, cerebrospinal fluid; IV, intravenous; IVT, intraventricular; PK, Pharmacokinetic; NA, not available.

Appendix 2. Clinical or loboratory response of treatment with intravenous or intraventricular vancomycin

| Author,  year | Administration | Study description | Multi-center / Single-center | Indication | Antimicrobial regimen | |  | Sample Size^a^ | CSF sterilization rate | | Adverse effects | Mortality |
| --- | --- | --- | --- | --- | --- | --- | --- | --- | --- | --- | --- | --- |
|  |  |  |  |  | Intervention | Comparator |  |  | Intervention | Comparator |  |  |
| Taheri, 2018 | IV | RCT | Single-center | PNM | VAN (CI group) | VAN (II group) |  | 20 (10/10) | 10 (100%) | 10(100%) | None | 0/20 (0.00%) |
| Elyasi, 2015 | IV | RCT | Single-center | BM | VAN (high-dose group) | VAN (conventional-dose group) |  | 44 (22/22) | NA | NA | None | 0/44 (0.00%) |
| Sipahi, 2013 | IV | Retrospective cohort study | Single-center | PNM and VPS | VAN | Linezolid |  | 17 (8/9) | 2/8 (25.0%) | 7/9 (77.78%) | NA | 9/17 (52.94%) |
| Arda, 2005 | IV | Retrospective study | Single-center | PNM and HAM | VAN | Teicoplanin |  | 6 (2/4) | NA | NA | NA | 0/6 (0.00%) |
| Lewin, 2019 | IVT | Retrospective cohort study | Multi-center | CNS infections | VAN | Aminoglycosides |  | 92 (44/48) | 39/44（88.4%） | 45/48（93.4%） | NA | 19/105 (18.1%) |
| Pfausler, 2003 | IV & IVT | RCT | Single-center | VPS | VAN (IVT group) | VAN (IV group) |  | 10 (5/5) | 5 (100%) | 5 (100%) | None | 0/10 (0.00%) |

^a^Expressed as total number of patients (number in Intervention group/number in comparator group). Abbreviation: CI, Continuous infusion group; CSF, cerebrospinal fluid; II, Intermittent infusion group; NA, not available; RCT, randomized controlled tria; VAN, Vancomycin.

Appendix 3. The risk of bias of included Randomized Controlled Trials

| Study | Random sequence generation (selection bias) | Allocation concealment (selection bias) | Blinding of participants and personnel (performance bias) | Blinding of outcome assessment (detection bias) | Incomplete outcome data (attrition bias) | Selective reporting (reporting bias) | Other bias |
| --- | --- | --- | --- | --- | --- | --- | --- |
| Taheri, 2018 | L | U | U | U | U | U | U |
| Elyasi, 2015 | L | U | U | U | U | U | U |
| Pfausler, 2003 | L | U | U | U | U | U | U |

Abbreviation: H, High risk; L, Low risk; U,Unclear risk.

Appendix 4. The quality of included Case-Control Studies

| Study | Is the Case Definition Adequate? | Representativeness of the Cases | Selection of Controls | Definition of Controls | Comparability of Cases and Controls | Ascertainment of Exposure | Use the same method to determine case and control exposure factors | Non-Response Rate | Total |
| --- | --- | --- | --- | --- | --- | --- | --- | --- | --- |
| Lewin, 2019 | ★ | ★ |  |  | ★ | ★ | ★ |  | 5 |
| Sipahi, 2013 | ★ | ★ |  | ★ | ★★ | ★ | ★ |  | 7 |
| Arda, 2005 | ★ | ★ |  |  |  | ★ | ★ |  | 4 |

Appendix 5. Quality assessment of included pharmacokinetic and TDM studies using the ClinPK statement

| No. | 1 | 2 | 3 | 4 | 5 | 6 | 7 | 8 | 9 | 13 | 10 | 11 | 12 | 14 |
| --- | --- | --- | --- | --- | --- | --- | --- | --- | --- | --- | --- | --- | --- | --- |
| Author, Year | Cai, 2019 | Taheri, 2018 | Parasuraman, 2018 | Wang, 2017 | Mounier, 2017 | Popa, 2016 | Lin, 2016 | Autmizguine, 2014 | Shokouhi, 2014 | Ricard, 2007 | Bafeltowska, 2004 | Albanèse, 2000 | Pfausler, 1997 | Viladrich, 1991 |
| 1 Title | ✓ | ✓ | ✓ | ✓ | ✓ | ✓ | ✓ | ✓ | ✓ | ✓ | ✓ | ✓ | ✓ | ✓ |
| 2 Abstract | ✓ | ✓ | ✓ | ✓ | ✓ | ✓ | ✓ | ✓ | ✓ | ✓ | ✓ | ✓ | ✓ | ✓ |
| 3 PK background | ✓ | ✓ | ✓ | ✓ | X | ✓ | ✓ | ✓ | ✓ | ✓ | ✓ | ✓ | X | X |
| 4 Study rationale | ✓ | ✓ | ✓ | ✓ | ✓ | ✓ | ✓ | ✓ | ✓ | ✓ | ✓ | ✓ | ✓ | ✓ |
| 5 Specific objective or hypothesis | ✓ | ✓ | ✓ | ✓ | ✓ | ✓ | ✓ | X | ✓ | ✓ | ✓ | ✓ | ✓ | ✓ |
| 6 Eligibility criteria of participants | ✓ | ✓ | ✓ | ✓ | ✓ | ✓ | ✓ | X | ✓ | ✓ | ✓ | ✓ | X | ✓ |
| 7 Coadministration of drugs/food | X | X | ✓ | ✓ | X | X | ✓ | X | ✓ | ✓ | X | X | X | ✓ |
| 8 Drug preparation and administration | ✓ | ✓ | ✓ | ✓ | ✓ | ✓ | ✓ | ✓ | ✓ | ✓ | X | ✓ | ✓ | ✓ |
| 9 Sampling described | ✓ | ✓ | ✓ | ✓ | ✓ | ✓ | ✓ | ✓ | ✓ | ✓ | ✓ | ✓ | ✓ | ✓ |
| 10 Validation of bioanalytical methods | ✓ | ✓ | ✓ | X | ✓ | X | ✓ | ✓ | ✓ | X | ✓ | X | ✓ | X |
| 11 PK modeling described | X | ✓ | X | X | X | X | ✓ | ✓ | X | X | X | ✓ | X | X |
| 12 Population PK model covariates described | X | X | X | X | X | X | ✓ | X | X | X | X | X | X | X |
| 13 Formulas provided | X | X | X | X | X | X | ✓ | X | X | X | X | X | X | X |
| 14 Body weight used | ✓ | X | X | X | X | X | ✓ | ✓ | X | X | X | X | X | X |
| 15 Statistical methods described | ✓ | ✓ | ✓ | ✓ | X | ✓ | ✓ | ✓ | ✓ | ✓ | X | X | ✓ | X |
| 16 Study withdrawals reported | X | ✓ | NA | NA | NA | NA | NA | NA | NA | NA | NA | NA | NA | ✓ |
| 17 Missing data quantified | X | ✓ | NA | NA | NA | NA | NA | NA | NA | NA | NA | NA | NA | NA |
| 18 Other explanatory variables provided | ✓ | X | X | X | X | X | ✓ | X | X | X | X | X | X | X |
| 19 Results reported with precision | ✓ | ✓ | ✓ | ✓ | X | ✓ | ✓ | ✓ | ✓ | ✓ | ✓ | ✓ | X | ✓ |
| 20 Dialysis methods described | NA | NA | NA | NA | NA | NA | NA | NA | NA | NA | NA | NA | NA | NA |
| 21 Bioavailability | NA | NA | X | NA | NA | NA | NA | NA | NA | NA | NA | NA | NA | NA |
| 22 Limitations described | ✓ | ✓ | ✓ | ✓ | X | X | ✓ | X | ✓ | X | ✓ | X | X | X |
| 23 Relevance of findings described | ✓ | ✓ | ✓ | ✓ | ✓ | ✓ | ✓ | ✓ | ✓ | ✓ | ✓ | X | X | ✓ |
| 24 Funding and author COI described | X/✓ | X | X/✓ | X | X | ✓ | ✓ | X | ✓ | X/✓ | ✓/X | X | X | X |

Abbreviation: COI, conflict of interest; NA, not applicable; PK, pharmacokinetic.
